# Optimization of Ultrasound-Assisted Extraction Using Response Surface Methodology for Simultaneous Quantitation of Six Flavonoids in Flos Sophorae Immaturus and Antioxidant Activity

**DOI:** 10.3390/molecules25081767

**Published:** 2020-04-12

**Authors:** Sanhong Fan, Gege Yang, Jinhua Zhang, Jiani Li, Baoqing Bai

**Affiliations:** 1 College of Life Science, Shanxi University, Taiyuan 030000, China; yanggege94@foxmail.com (G.Y.);; 2 Shanxi Key Laboratory for Research and Development of Regional Plants, Taiyuan 030000, China

**Keywords:** flos sophorae immaturus, flavonoids, ultrasound-assisted extraction, HPLC, antioxidant activity

## Abstract

Ultrasound-assisted extraction (UAE) was applied to extract rutin (RU), nicotiflorin (NI), narcissoside (NA), kaempferol (KA), isorhamnetin (IS), quercetin (QU), and total flavonoids of Flos Sophorae Immaturus (TFFSI) from Flos Sophorae Immaturus (FSI). Through single factor test and response surface methodology (RSM), the optimal extraction conditions were concluded as follows: ethanol concentration 70%, time 30 min, temperature 61 °C, and liquid/solid ratio 15.30 mL/g, respectively. The actual extraction rates of RU, NI, NA, KA, IS, QU, and TFFSI were 14.6101%, 2.9310%, 7.1987%, 0.1041%, 0.4920%, 2.7998%, and 26.4260%, respectively. The experimental results demonstrated that the extraction method with accuracy and efficiency could be used for the comprehensive evaluation quality control of extracts from FSI. The antioxidant activities of hydroalcoholic extraction from FSI on 1,1-diphenyl-2-picrylhydrazyl (DPPH), 2,2′-azino-bis (3-ethylbenzothiazoline-6-sulfonic acid) (ABTS•^+^), superoxide anion (•O^2−^) free radicals, and ferric reducing/antioxidant power (FRAP) were assessed. The results showed that the antioxidation activities of extracts on DPPH, ABTS•^+^, and •O^2−^ free radicals were reached 89.29%, 97.86%, and 56.61%, and 81.4% in FRAP at 1.0 mg/mL, respectively. The antioxidant capacity of FSI extract was positively correlated with the amount of total flavonoids.

## 1. Introduction

Flos Sophorae Immaturus (FSI) is the dried flower bud of leguminous plant *Sophora japonica L.* that is widely cultivated in many provinces of China, Japan, and Korea, which is a valuable Chinese herbal medicine with heat-clearing, cooling blood, and hemostasis [1]. Modern pharmacological studies have shown that the efficacy of FSI is closely related to its main component-flavonoids. Researches have shown that flavonoids from FSI have many potential physiological activities, such as brain protection [2,3], cardiovascular protection [4], liver protection [5], anti-cancer [6], anti-platelet aggregation [7], anti-inflammatory [8,9], anti-allergy [10], and anti-oxidation [11]. With the in-depth study of the ingredients of flavonoids from FSI, it has been found that FSI contains rutin (RU), narcissoside (NA), nicotiflorin (NI), kaempferol (KA), quercetin (QU), isorhamnetin (IS), and genistein, which have their own pharmacological effects [12,13,14]. Therefore, it is very necessary to optimize the extraction procedure of flavonoids from FSI, and the qualitative and quantitative analysis of flavonoids was carried out.

At present, simultaneous extraction of several flavonoids’ compounds from FSI have been reported, as described in Table 1. Gen Wang et al. optimized the extraction conditions of RU, NI, NA, KA, IS, and QU from FSI by deep eutectic solvent (DES) using microwave-assisted extraction (MAE) [15]. In Jin-Liang Liu et al.′s study, they optimized the extraction process of RU, KA, IS, QU, and genistein from FSI by methanol using MAE [16]. Zhi-sheng Xie et al. optimized the extraction procedure of RU, NI, and NA from FSI by methanol using ultrasound-assisted extraction (UAE) [17]. In addition, Fa-jie Li et al. [18] and Zhi-bin Gan et al. [19] optimized the extraction conditions of RU from FSI by methanol using infrared-assisted extraction (IAE) and far infrared-assisted solvent extraction (FIASE), respectively. Among these extraction methods, UAE is the most convenient and mature technology; thus, it is widely used in industrial production to extract chemical components from Chinese herbal medicines [20]. UAE has the edge of high yield, low cost, simple operation, and high efficiency compared with those extraction methods [21,22]. Ultrasonic can create cavitation between the solvent and the sample, relying on bubble formation and their fierce collapse, which leads to the formation and explosion of bubbles in the solution [17]. It facilitates solute diffusion and improves the mass transfer rates of target compounds from solid phase to liquid phase by destroying the cell wall, thus enhancing yield in a shorter time [23]. The ultrasonic method can greatly reduce the extraction time and accelerate the extraction rate of active ingredients and utilization rate of raw materials [24]. Therefore, in this experiment, univariate experiments and response surface method (RSM) were utilized to optimize the extraction process of total flavonoids and six flavonoids from FSI by UAE.

On the other hand, solvent plays an important factor in the extraction of components from natural materials. Previous studies have reported the extraction flavonoids from FSI using ethanol, methanol, DES, ionic liquids (ILs), and 1-butyl-3-methylimidazoliumchloride (BMIM-Cl) solution, as shown in Table 1. However, methanol is neurotoxic, the preparation of ILs is more difficult and DES and BMIM-Cl in the laboratory research stage and cannot be mass-produced [15,25]. Therefore, an environment-friendly and efficient method to extract flavonoids from FSI is very important. It has far-reaching significance for the effective utilization of bioactive substances in FSI and the elucidation of their pharmacological effects.

The purpose of our article is to study the extraction process of flavonoids in FSI. The effects of the types of extraction organic solvent, the species of surfactants added in extraction solvent, the concentration (*v*/*v*) of extraction solvent, the liquid–solid ratio (mL/g), ultrasound extraction time (min), and ultrasound extraction temperature (°C) were optimized by RSM. More importantly, the antioxidant activity of flavonoids extracted by UAE was evaluated, which provided a theoretical basis for further exploration of their biological activity in the future.

## 2. Results and Discussion

### 2.1. Univariate Experiments Design and Analysis

#### 2.1.1. Effects of Solvent Type on UAE

**Figure 1:**
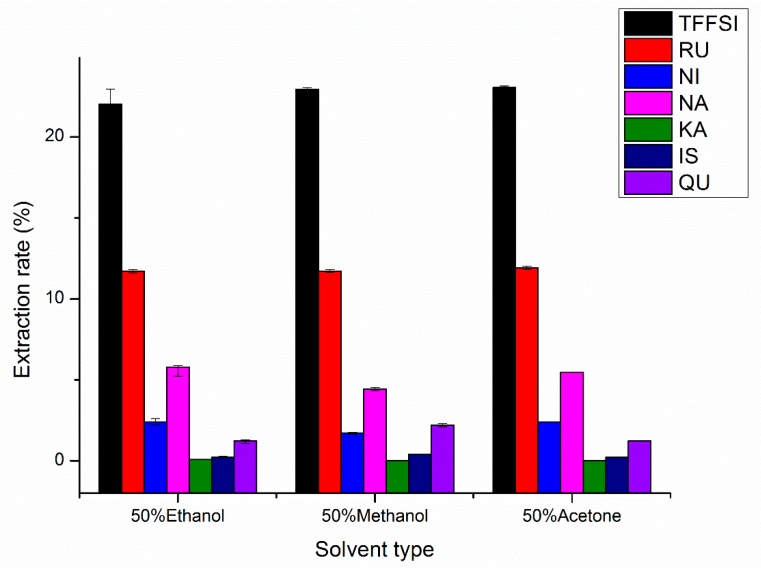
Effects of the solvent type on the extraction rates of rutin (RU), nicotiflorin (NI), narcissoside (NA), kaempferol (KA), isorhamnetin (IS), quercetin (QU), and the total flavonoids of Flos Sophorae Immaturus (TFFSI).

Solvent selection plays an important factor in the extraction of components from natural materials. [16]. Therefore, the appropriate solvent selection in the study can be accelerated the dissolution of the target compound. When the liquid–solid ratio, temperature, and extraction time were set as 15 mL/g, 60 °C, and 30 min, respectively, the effects of 50% ethanol, 50% methanol, and 50% acetone on the extraction efficiency of flavonoids were shown in Figure 1. However, there were insignificant differences in the extraction rates of total flavonoids of Flos Sophorae Immaturus (TFFSI), RU, NI, NA, KA, IS, and QU, when the solvent type was changed. Fifty percent acetone possessed an apparent advantage over other solvents in extracting TFFSI and RU from FSI. Fifty percent ethanol had a distinct advantage over other solvents in extracting NI, NA, and KA. Moreover, the definite advantage of 50% methanol is higher yields of IS and QU in FSI, similar to Jin-Liang Liu’s [16] results. Compared to ethanol, methanol and acetone are toxic (with the anesthetic effect to the central nervous system), expensive, and environmentally unfriendly [17]. Additionally, ethanol is an easily available solvent with fast, efficient, safe, green, sustainable, and has a high extraction rate [27]. Therefore, ethanol was chosen from the above the extraction solvents as the best appropriate extraction solvent.

#### 2.1.2. Effects of the Species of Surfactants on UAE

**Figure 2:**
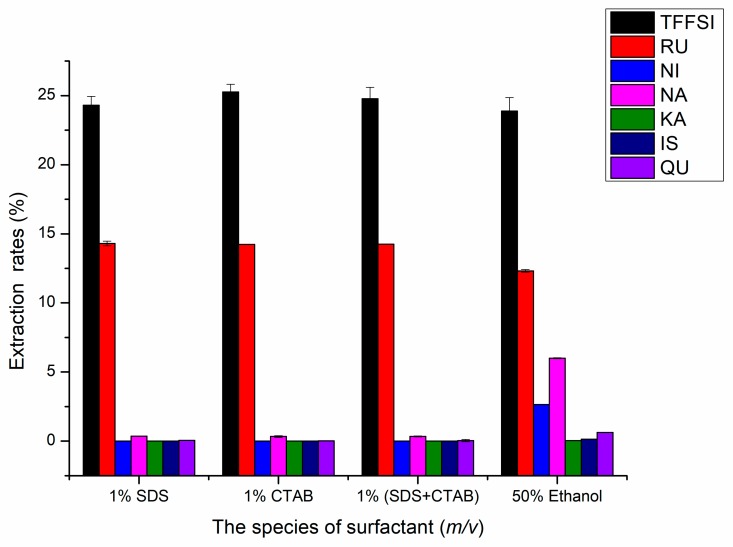
Effects of the species of surfactant on the extraction rates of RU, NI, NA, KA, IS, QU, and the TFFSI.

As a kind of food additive, surfactant has little toxicity, low residue, and environment-friendly, and can increase the dissolution and exudation ability of macromolecular organic substances [28]. Therefore, it has great application potential to use the solubilization effect of surfactant to co-extract natural active substances. There have been preliminary studies on the extraction of flavonoids. Based on the Zhu Xin et al. [29] research results, in this study, cationic surfactant-sodium dodecyl sulfate (SDS), anionic surfactants-cetyl trimethyl ammonium bromide (CTAB), and SDS:CTAB (ratio of 1:1) were selected, and the extraction effects of TFFSI and six flavonoids were discussed. The yields of TFFSI and six flavonoids by adding three different surfactants (1% (*m*/*v*) SDS, 1% (*m*/*v*) CTAB, and 1% (*m*/*v*) (SDS + CTAB)) were shown in Figure 2. Other experimental conditions were as listed below: Liquid-solid ratio 15 mL/g, extraction temperature 60 °C, ethanol concentration 50%, and extraction time 30 min. The result indicated that the extraction rates of TFFSI increased by 0.42%, 1.38%, and 0.88%, and the extraction rate of rutin increased by 1.98%, 1.91%, and 1.93%, respectively, compared with 50% Ethanol. The reason for the increase in extraction rates may be that the anionic and cationic surfactants are better associated with RU and TFFSI, forming the association structure, which increases the solubility [29]. While the extraction rates of NA, NI, and QU decreased significantly, compared with 50% ethanol, for 1% (*m*/*v*) SDS, 1% (*m*/*v*) CTAB, and 1% (*m*/*v*) (SDS + CTAB), the extraction rate of NA decreased by 5.63%, 5.66%, and 5.66%, the extraction rate of NI decreased by 2.65043%, 2.6505%, and 2.65042%, and the extraction rate of QU decreased by 0.565%, 0.598%, and 0.58%, respectively. Furthermore, KA and IS were not detected in experimental groups with three different surfactants. The cause is that the formed surfactant micelles combine with other substances, weakening the solubilization of the above flavonoids [30]. However, due to the rich foaming property of the surfactant, it is easy to suck back when the extracts were concentrated by rotary evaporation subsequently, which caused inconvenience to the subsequent experiments. In view of the improvement of extraction rates of TFFSI and six flavonoids was insignificant, so surfactant was abandoned to assist the extraction of flavonoids in FSI.

#### 2.1.3. Effects of the Ethanol Concentration on UAE

RU, NI, and NA are flavonoid glycosides with the larger polarity, and QU, KA, and IS are the corresponding flavonoid aglycones of the former, respectively, and their polarity is relatively small [15]. They are all planar molecules with tight molecular packing, large inter molecular attraction, and more hydrophilic hydroxyl groups, so the solubility in water is not large. As a green organic solvent to extract the target flavonoids, ethanol concentration determines the polarity of the extraction solvent, which determines the hydrophobic force and hydrogen bond bonding strength of the target components in the solvent and affecting the solubility and extraction rate of the target components [31]. Therefore, it is very important to research ethanol concentration in the experiment.

**Figure 3:**
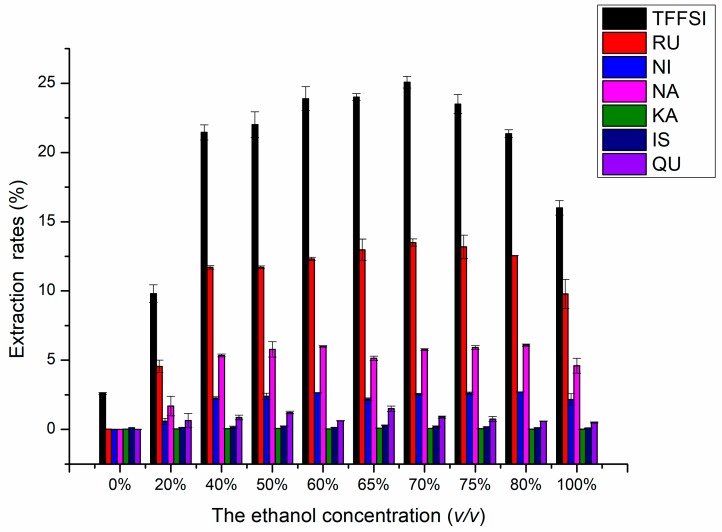
Effects of the ethanol concentration on the extraction rates of RU, NI, NA, KA, IS, QU, and the TFFSI.

The influence of the ethanol concentration on the rates of TFFSI and six compounds were shown in Figure 3. Extractions were carried out at different ethanol concentration of 0%, 20%, 40%, 50%, 60%, 65%, 70%, 75%, 80%, and 100% at liquid–solid ratio 15 mL/g, extraction temperature 60 °C, and extraction time 30 min. The rates of TFFSI, RU, NI, NA, KA, IS, and QU significantly increased when ethanol concentration ranged from 0% to 70%, and then lowered slightly with higher ethanol concentration. Following the theory of similarity and inter miscibility, the more similar the polarity of solvent and solute, the faster the dissolution of solute from plant cells [32]. When the ethanol concentration was 70%, the highest yields (TFFSI, RU, NI, NA, KA, IS, and QU) were 25.08%, 13.50%, 2.70%, 6.10%, 0.09%, 0.30%, and 1.50%, respectively. The mechanism may be that as the concentration of ethanol increases, the target component is combined with ethanol through hydrogen bond or van der Waals force, so as to improve the dissolution and extraction rate. When the ethanol concentration was greater than 70%, the extraction rate of the target component decreased slightly. The reason may be that the denaturation of protein increases the diffusion resistance at higher ethanol concentration [33]. To sum up, 70% ethanol concentration was chosen as subsequent experiments.

#### 2.1.4. Effects of Liquid-Solid Ratio on UAE

**Figure 4:**
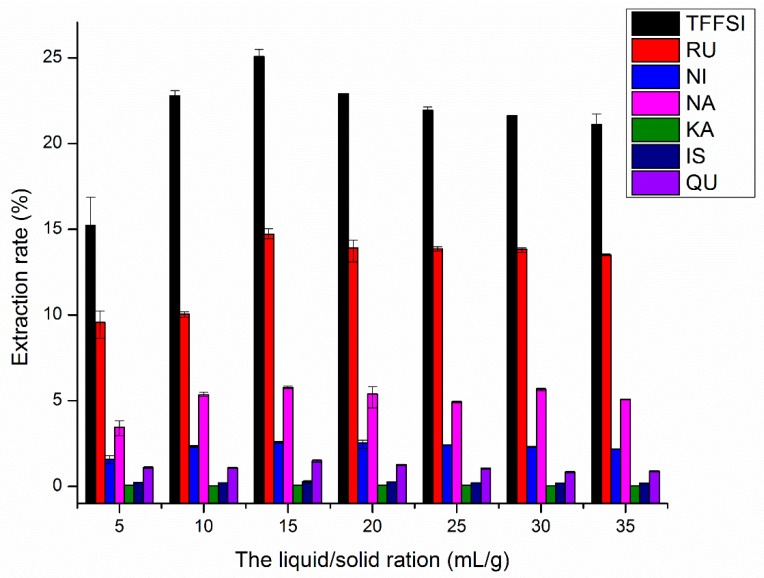
Effects of liquid–solid ratio on the extraction rates of RU, NI, NA, KA, IS, QU, and the TFFSI.

Different liquid–solid ratio could significantly affect the extraction yield. When the amount of extractant is insufficient, which will lead to incomplete extraction. However, when the solvent is redundant, which may also cause lower extraction yields and solvent waste [16]. Thus, the liquid-to-solid ratio must be appropriate [34]. The effect of different liquid–solid ratio at the 70% ethanol concentration, extraction time 30 min and extraction temperature 60.0 °C was drawn in Figure 4. The yields of TFFSI and six flavonoids increased significantly with increasing liquid–solid ratio from 5.0 to 15.0 mL/g, the yield reached a maximum at 15.0 mL/g, with the extraction rates of TFFSI, RU, NI, NA, KA, IS, and QU were 25.07%, 14.72%, 2.56%, 5.77%, 0.080%, 0.29%, and 1.49%, respectively. The reason may be that the improvement of the mass transfer rates and diffusion of the flavonoids compounds along with the rise of the liquid–solid ratio [18]. However, when the ratio continued to increase (> 15 mL/g), the yields decreased a little. Due to the mechanical effect and pyrometric effect being produced by the ultrasonic wave, it began to become no longer significant, but promoted the dissolution of other impurities and antagonized the target component, so that the extraction rate of flavonoids began to decrease instead of increasing. These results were consistent with the previous studies [16]. Therefore, the liquid–solid ratio of 15.0 mL/g is beneficial for the extraction of flavonoids.

#### 2.1.5. Effects of Ultrasound Extraction Time on UAE

**Figure 5:**
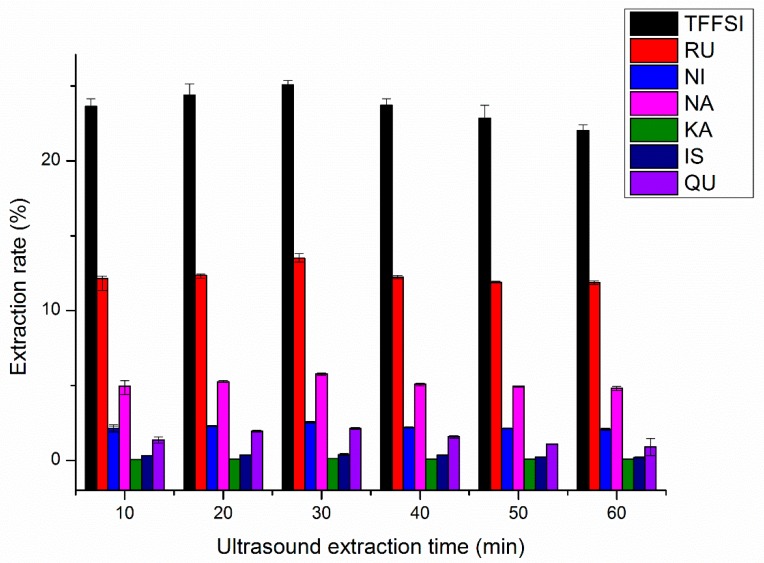
Effects of the ultrasound extraction time on the extraction rates of RU, NI, NA, KA, IS, QU, and the TFFSI.

Extraction time was another parameter that would affect the extraction yield. Sufficient extraction time can improve the interaction between the extraction agent and the FSI powder, which may accelerate the solubility of target compounds and facilitate extraction [34]. However, the longer the extraction time, the higher the risk of oxidation of flavonoids, which may lead to lower processing potency and high energy consumption [16]. Therefore, during the extraction process, the medium extraction time is usually to reduce the oxidation of flavonoids. Extraction was performed at the length of different extraction times while other parameters were fixed as follows: The ethanol concentration, liquid–solid ratio, and extraction temperature were 70%, 15.0 mL/g, and 60.0 °C, respectively. The influences of extraction time on the extraction rates of TFFSI and the six flavonoids from FSI were indicated in Figure 5. The extraction rates of TFFSI and six flavonoids were increased with extraction time and reached its maximum at 30 min. The maximum extraction rates (TFFSI, RU, NI, NA, KA, IS, and QU) were 25.09%, 13.50%, 2.55%, 5.77%, 0.11%, 0.39%, and 2.13%, respectively. The results might be the time requirement of the FSI powder cytoderm to break by ultrasonic wave, and the solvent to permeate into the dried powders and dissolve the flavonoids [34]. The flavonoids yield decreased slightly after 30 min (Figure 5). It is inferred that long extraction time induced the degradation of flavonoids dissolved in solution, and flavonoids may be destroyed by the huge mechanical energy generated by ultrasonic wave, the yield of flavonoids decreased [16]. Therefore, the ultrasonic extraction time of 30 min was considered to be optimal in the current experiment.

#### 2.1.6. Effects of Ultrasound Extraction Temperature on UAE 

**Figure 6:**
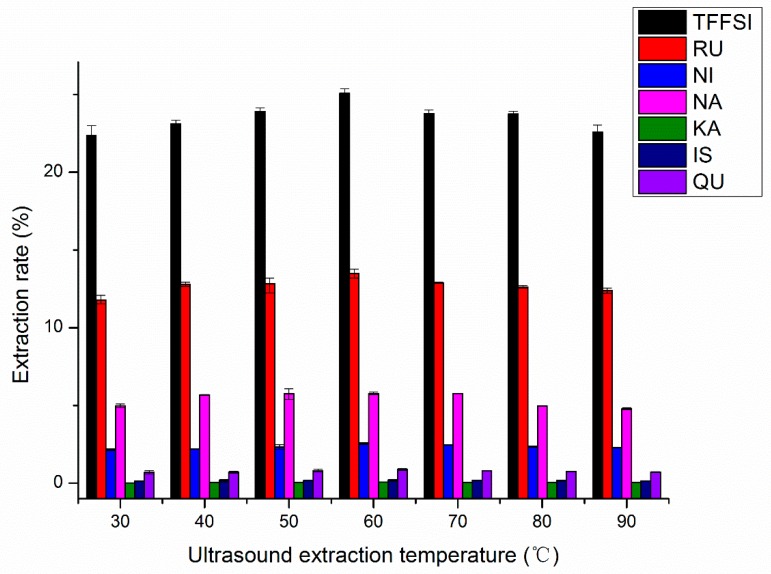
Effects of the ultrasound extraction temperature on the extraction rates of RU, NI, NA, KA, IS, QU, and the TFFSI.

The appropriate extraction temperature is an important factor in the optimization of the extraction process. With the increase of temperature, the stickiness and surface tension of the solvent decreased, and the solubility and diffusion rate of flavonoids are increased, so the extraction rate of flavonoids is increased. However, higher temperature leads to a decline of the extraction rate of flavonoids due to thermal degradation [34]. As shown in Figure 6, the effect of temperature on extraction yield was assessed. While other extraction conditions were adjusted as follows: The ethanol concentration 70%, liquid–solid ratio 15.0 mL/g, and extraction time 30 min, the yields of TFFSI, RU, NI, NA, KA, IS, and QU significantly were increased when extraction temperature changed from 30.0 to 60.0 °C. The maximum yields of TFFFSI, RU, NI, NA, KA, IS, and QU were observed when extraction temperature was 60.0 °C, they were 25.08%, 13.50%, 2.55%, 5.77%, 0.078%, 0.197%, 0.06%, and 0.89%, respectively. It may be that appropriate heat treatment reduces the viscosity of the solvent, the molecular movement speed in the material increases gradually, which is conducive to the diffusion of the solute in the extraction system, thus enhancing the solubility of the flavonoids in the solution [16]. When the ultrasonic extraction temperature exceeded 60 °C, the high temperature destroyed the structure of flavonoids, made flavonoids denaturation and reduced the extraction rate of the target flavonoids. This result is consistent with the earlier observation [16]. Therefore, extraction temperature of 60.0 °C was selected as the optimal option in the present experiment.

### 2.2. Multi-Response Design and Analysis

#### 2.2.1. Model Fitting

**Figure 7:**
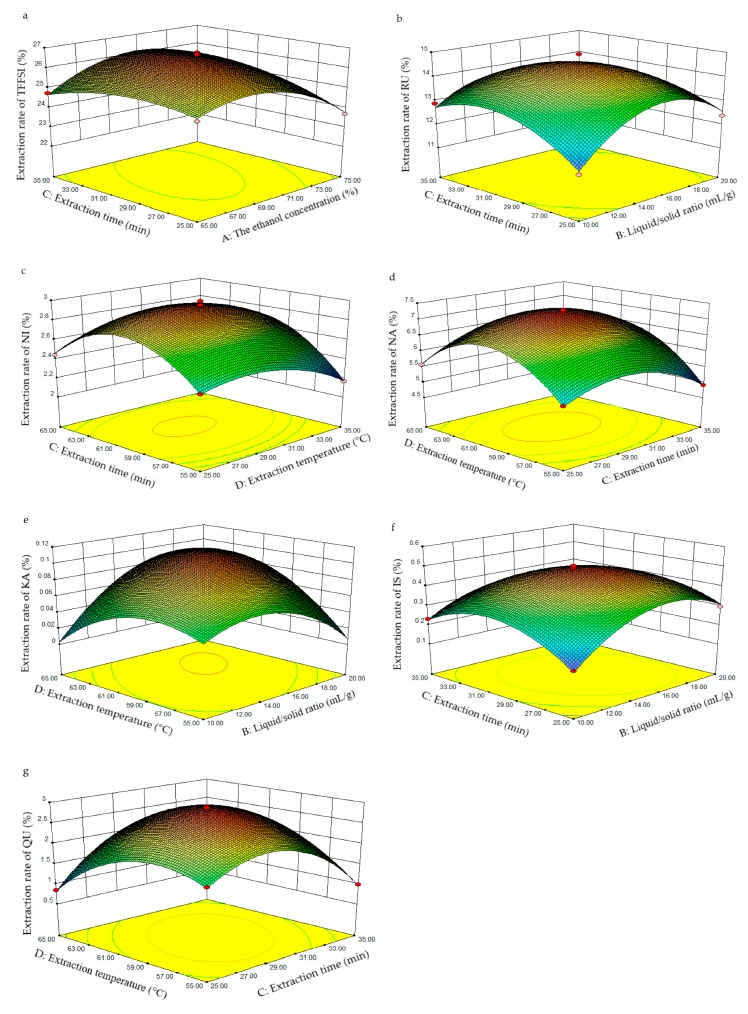
The response surfaces of the TFFSI (**a**), RU (**b**), NI (**c**), NA (**d**), KA (**e**), IS (**f**), and QU (**g**) from Flos Sophorae Immaturus: (**a**) varying the ethanol concentration and ultrasound extraction time; (**e**) varying liquid/solid ratio and ultrasound extraction temperature; (**b** and **f**) varying liquid/solid ratio and ultrasound extraction time; and (**c**, **d**, and **g**) varying ultrasound extraction temperature and ultrasound extraction time.

On the ground of the outcomes of univariate experiments, four main extraction conditions (the ethanol concentration, liquid/solid ratio, ultrasound extraction time, and ultrasound extraction temperature) of extraction of flavonoids from FSI were further optimized by BBD. According to previous experiments, the extent of four pivotal extraction parameters were identified as follows: The ethanol concentration (65%–75%), liquid/solid ratio (10–20 mL/g), ultrasound extraction time (25–35 min), and ultrasound extraction temperature (55–65 °C), respectively. To optimize extraction process, a twenty-nine-run BBD with four process parameters and three levels (Table 2) were performed, and the extraction rates of TFFSI and six flavonoids (RU, NI, NA, KA, IS, and QU) were taken as the dependent variables (Figure 7). Based on the multivariable regression analysis model, the quadratic polynomial equation of TFFSI and six flavonoids were measured as follows:
Y_1_ = 26.35−0.19A + 0.57B + 0.27C + 0.34D + 0.14AB + 0.53AC−0.20AD + 1.04BC + 0.39BD + 0.53CD−1.11A^2^−1.81B^2^−0.50C^2^−1.45D^2^ Y_2_ = 14.52 + 0.11A−4.941E−0.004B + 0.079C + 0.032D−0.45AB−0.16AC + 0.020AD−0.46BC + 0.27BD + 0.27CD−1.03 A^2^−1.39 B^2^−0.95 C^2^−1.02 D^2^ Y_3_ = 2.92 + 0.049A + 7.798E−0.003B−0.040C + 0.11D−0.011AB + 0.043AC + 1.688E−003AD + 0.025BC + 0.018BD + 0.079CD−0.19 A^2^−0.19 B^2^−0.17 C^2^−0.35 D^2^ Y_4_ = 7.22 + 0.079A−0.044B−0.085C + 0.26D−0.010AB + 0.084AC + 0.054AD + 0.048BC−0.018BD + 0.16CD−0.74 A^2^−0.73 B^2^−0.68 C^2^−1.13 D^2^ Y_5_ = 0.10 + 5.705E−0.003A + 0.012B + 6.440E−003C + 0.010D + 9.904E−003AB−4.594E−003AC + 6.230E−003AD + 4.234E−003BC + 0.031BD + 0.012CD−0.050 A^2^−0.034 B^2^−0.051 C^2^−0.03 D^2^ Y_6_ = 0.49 + 2.864E−003A + 0.031B−0.010C + 0.022D + 0.016AB + 5.471E−003AC + 0.022AD−0.044BC−1.988E−003BD−0.017CD−0.12 A^2^−0.14 B^2^−0.13 C^2^−0.085 D^2^ Y_7_ = 2.85−0.13A + 0.026B + 0.026C−0.046D−1.566E−003AB−0.030AC−7.115E−003AD−0.094BC−0.20BD + 0.45CD−0.86 A^2^−0.93 B^2^−0.85 C^2^−0.66 D^2^

The process of hydroalcoholic extraction of flavonoids from FSI can be speculated and optimized through the regression equation. Where Y_1_, Y_2_, Y_3_, Y_4_, Y_5_, Y_6_, and Y_7_ were the extraction rates of TFFSI, RU, NI, NA, KA, IS, and QU, respectively; A was the ethanol concentration, B was liquid/solid ratio (mL/g), C was ultrasound extraction time (min), and D was ultrasound extraction temperature (°C). The linear coefficients of four parameters testify that the rank of variables affecting the extraction rates of flavonoids. The larger the number, the higher the rank. The positive sign of linear showed that the dependent variable is increasingly correlated with the independent variable, and vice versa. Moreover, positive quadratic coefficient demonstrated that the opening of equation curve was upward, which concluded that it had minimum values and could not conduct optimal analysis. However, when the opening curve was downward, we could obtain the optimal values of extraction rates. By comparing the figures, we found that the order was as follows: Liquid/solid ratio > extraction temperature > extraction time > the ethanol concentration for TFFSI. The other six flavonoids also like that.

The analysis of variance (ANOVA), goodness-of-fit, and relevance of the regression model were concluded in Table 2. Statistical ANOVA was used to predict the interaction among variables in the optimization hydroalcoholic extraction of flavonoids from FSI. The *p*-value in the *F*-test was used to measure the significance level of variables. Specifically, when *p* <0.05, the model is statistically significant, and when *p* <0.01 the model is extremely significant. The *F*-values (> 48.48) and *p*-values (<0.0001) of the model for TFFSI, RU, NI, NA, KA, IS, and QU indicated that seven groups of models were extremely statistical significance and the experimental errors were minimal and the results showed that the model was correct to analyze data. Values of “Lack of fit *F*-value” are less than 0.89 and values of “Lack of fit *p*-value” were more than 0.6025. Those results showed that the lack of fit was not of statistical significance, and the random error and the systematic error had no remarkable effect on the model and the regression data can be accurately displayed through the founded model.

Furthermore, the correlation coefficient (*R^2^*) and their adjusted *R^2^* are applied to evaluate the reasonability and feasibility of the fitting. For TFFSI, RU, NI, NA, KA, IS, and QU, the correlation coefficient (*R^2^*) values exceeded 0.9798, and the adjusted *R^2^* exceeded 0.9596, which manifested that the measured data matched well with the prediction value. The outcomes of ANOVA also indicated the statistical significance of the first degree terms A, B, C, and D, interaction terms AB, AC, AD, BC, BD, and CD, and second-order terms A^2^, B^2^, C^2^, and D^2^, respectively, which indicated that the influence of each factor on the response was both linear and interactive. The *F*-value reflects the contribution rate of each variable to the response value. The higher the *F*-value, the greater the influence of the independent variable on the extraction rate of the flavonoids from FSI. It may be seen from the *F*-values, for TFFSI, RU, NI, NA, KA, IS, and QU the order of factors affecting the extraction rate were B > D > C > A, A > C > D > B, D > A > C > B, D > C > A > B, B > D > C > A, B > D > C > A, and A > D > C > B, respectively (Table 2). For TFFSI; A, B, C, D, AC, BC, BD, CD, A^2^, B^2^, C^2^, and D^2^ were extremely significant model items, and AD was vital model items. For RU; AB, BC, A^2^, B^2^, C^2^ and D^2^ were extremely significant model items, and BD and CD were significant model items. For NI, A, C, D, CD, A^2^, B^2^, C^2^, and D^2^ were extremely significant model items, and AC was notable model items. For NA; A, B, C, D, AC, CD, A^2^, B^2^, C^2^, and D^2^ were extremely significant model items, and AD and BC were notable model items. For KA; A, B, C, D, AB, BD, CD, A^2^, B^2^, C^2^, and D^2^ were extremely significant model items, and AD was remarkable model items. For IS; B, D, BC, A^2^, B^2^, C^2^, and D^2^ were extremely significant model items, and C, AD, and CD were important model items. For QU; A, D, BC, BD, CD, A^2^, B^2^, C^2^, and D^2^ were extremely significant model items, and B and C were notable model items (Table 2). Therefore, four factors possessed a remarkable influence on the extraction rates of TFFSI and six compounds.

#### 2.2.2. Interpretation of Response Surface Models

Response surfaces (three-dimensional) were drawn by Design-Expert software (8.0.6) to elucidate the interactions of factors for the highest response value. Each figure showed the effects of two factors on the extraction yields of target components while the third factor and the fourth factor were fixed at zero levels. The influences of the interaction of the four parameters on the extraction rates of TFFSI and the six flavonoids were clarified through three-dimensional (3D) response surface plots (Figure 7). The mutual effect of the ethanol concentration and ultrasound extraction time on the extraction rate of TFFSI was investigated (Figure 7a). With the rise of the ethanol concentration and ultrasound extraction time, the extraction rate of TFFSI also improved gently. Whereas, the extraction rate of TFFSI began to decline as the ethanol concentration was more than 70%. It could be that the denaturation of proteins in higher ethanol concentrations increased diffusion resistance [33]. With the growth of liquid–solid ratio and ultrasound extraction time, the extraction rates of RU and IS rose increasingly (Figure 7b,f). When the time and liquid–solid ratios were more than 30 min and 16 mL/g, the extraction rates of RU and IS began to reduce, and related literature had reported that redundant solvent could lead to a decrease in the extraction yields of target compounds [35]. The extraction rates of NI, NA, and QU also raised with the rise of ultrasound extraction time and temperature (Figure 7c,d,g). When the time and temperature were more than 30 min and 61 °C, the extraction rates of NI, NA, and QU were increased slightly. It may be that prolonged high temperatures lead to the breakdown of flavonoid compounds [16]. As the growth of the liquid–solid ratio and ultrasound extraction temperature, the extraction rate of KA went up rapidly (Figure 7e). When the temperature exceeded 61 °C, the extraction effect decreased significantly. In summary, the higher extraction rates of six flavonoids can be achieved by changing the extraction conditions.

#### 2.2.3. Verification of Predictive Models

Simultaneous optimizations of the multiple dependent variables were carried out using Derringer′s desirability function method. The entire desirability value for the chosen optimum design was 0.940. The optimal process parameters were ethanol concentration, liquid–solid ratio, time and temperature of 70.10%, 15.36 mL/g, 30.13 min, and 60.53 °C, the predicted top extraction rates (TFFSI, RU, NI, NA, KA, IS, and QU) were 26.4117%, 14.5128%, 2.92494%, 7.22633%, 0.10358%, 0.487876%, and 2.83639%. In consideration of the convenience in operation, the parameters of the ethanol concentration, liquid–solid ratio, ultrasound extraction time, and ultrasound extraction temperature were revised to 70%, 15.30 mL/g, 30 min, and 61 °C, respectively. The actual extraction rates of TFFSI, RU, NI, NA, KA, IS, and QU were 26.4260%, 14.6101%, 2.9310%, 7.1987%, 0.1041%, 0.4920%, and 2.7998%, respectively, with relative standard deviation (RSD) values lower than 0.92%. All experiments were performed in triplicate. It was verified that the results of the verification experiments were similar to the predicted values acquired from the optimization models.

### 2.3. Method Validation

The linearity, the limit of detection (LOD), and the limit of quantification (LOQ) were verified in our study. The linearity was founded through the assessment of gradient concentrations of the standard solution. All processes were performed repeatedly three times. The least-square linear regression model had established the standard curve between the concentration (X) of the rutin standard solution and the matching UV absorbance value (Y) for TFFSI. Furthermore, the calibration curves between the concentration (X) of six flavonoids standards solution and the matching peak area (Y) were set up by the linear regression model for RU, NI, NA, KA, IS, and QU (Table 3). When the signal-to-noise ratios (S/N) of analytes were 10:1 and 3:1, the concentrations of analytical samples were LOQ and LOD. The correlation coefficients of all seven response values exceeded 0.991, and the values of LOQ and LOD were very low (Table 3). The above results show that the analysis of target compounds has high sensitivity.

The precision, repeatability, and recovery on experiment data were illustrated in Table 4. Six successive injections of mixed standard solutions were performed, and the repeatability was determined by measuring the relative standard deviation (RSD) of retention time (Rt) and peak area (Pa). The RSD values of Pa and Rt were changed from 0.04% to 0.91% and from 0.04% to 0.60%, respectively. Precision was examined through the RSD values of Pa and Rt at intra-day and inter-day. Intra-day RSD of Pa and Rt varied from 0.13% to 0.89% and from 0.24% to 0.95%, and inter-day RSD of Pa and Rt ranged from 0.05% to 0.70% and from 0.09% to 0.62%. The recovery was investigated by adding a certain amount of standard solution into the known contents FSI sample solution. The recoveries of the six flavonoids were between 97.68% and 99.41%, and the RSD values were between 2.14% and 3.16%. All the data indicated that the HPLC method was appropriate for the quantity and quality analysis of six flavonoids in FSI.

### 2.4. Assess the Antioxidant Activity

The antioxidant capacity has relation to many opportunities, such as the structure of the flavonoids, other components in the flavonoid sample [36]. In our study, the antioxidant activities of FSI extract were carried out via four different methods: 1,1-diphenyl-2-picrylhydrazyl (DPPH), 2,2′-azino-bis (3-ethylbenzothiazoline-6-sulfonic acid) (ABTS•^+^), superoxide anion (•O_2_^−^), and ferric reducing/antioxidant power (FRAP). These experiments were analyzed with the standard rutin, respectively. Meanwhile the standard antioxidant of vitamin C (VC) was compared.

#### 2.4.1. Scavenging Capacity of DPPH Radical

**Figure 8:**
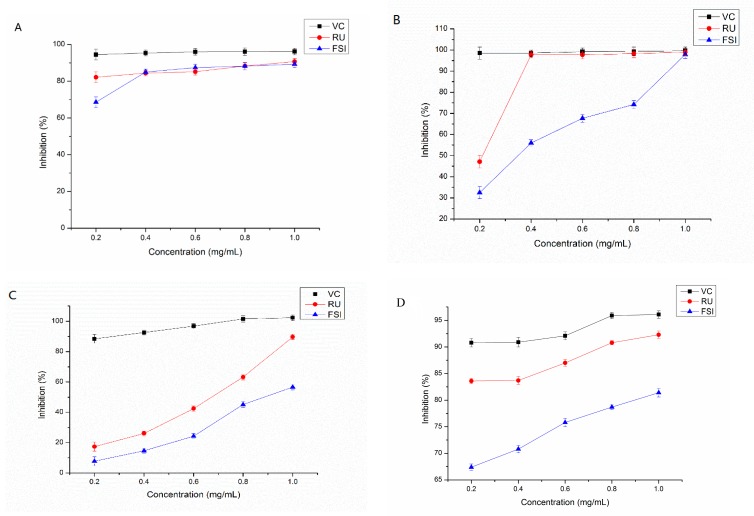
Effect of gradient concentration of FSI extracts of in antioxidant capacity tests: (**A**) 1,1-diphenyl-2-picrylhydrazyl (DPPH) assay, (**B**) 2,2′-azino-bis (3-ethylbenzothiazoline-6-sulfonic acid) (ABTS•^+^) assay, (**C**) superoxide anion assay, and (**D**) reducing power assay. (VC: Vitamin C).

It is generally known that DPPH is a steady free radical. Compared with other methods, it could assess the antioxidant capacity in a short time [37]. DPPH can be dissolved in ethanol, and has typical absorption at 517 nm. When the antioxidant is added, the colors of DPPH assay solutions fade [38]. In Figure 8A, the inhibition of the DPPH radical on FSI hydroalcoholic extracts was stated. The DPPH radical inhibition capacity (y) of the FSI extract display a second-order dependence on concentration (x, 0.0–1.0 mg/mL) (*p* <0.05), and can be summed up by the equations y = −57.565x^2^ + 91.361x + 54.211 (*R^2^* = 0.9252). The results showed that FSI hydroalcoholic extracts extract could directly scavenge the stable DPPH radicals in the concentration extent of 0–1.0 mg/mL, and the maximum inhibition rate was 89.29% at 1.0 mg/mL. When the concentration of FSI extract was greater than 0.4 mg/mL, the scavenging activity of DPPH radical tends to be stable. In the concentration extent 0.4–0.8 mg/mL the DPPH inhibition percentage values of FSI extract were slightly higher than that of the rutin standard, which was consistent with the results of the FSI extracts reported by Wang et al. [39]. The former was slightly below the latter at 1.0 mg/mL, which may be the result of experimental error. Overall, FSI extracts have a better scavenging capacity of DPPH radicals in the range of the tested concentration.

#### 2.4.2. Scavenging Capacity of ABTS•^+^

ABTS•^+^ could generally assess the antioxidant activity of bioactive compounds with the discoloration reaction [37]. As figured in Figure 8B, the ABTS•^+^ inhibition activities were related to their concentrations, which increased in the extent of the tested concentrations, for the FSI extracts and the positive control (VC and RU). The ABTS•^+^ inhibition capacity (y) and concentration (x) of the FSI extract was associated by the following second-order equations, respectively: y = −8.6488x^2^ + 84.88x + 18.519 (*R^2^* = 0.9623). The highest clearance rate reached 97.86%. The ABTS•^+^ inhibition decreased in the order VC > RU > FSI. When the concentrations are low (<0.40 mg/mL), VC presented the best antioxidant capacity. When the concentration from 0.4−1.0 mg/mL, the scavenging ability of RU standard to ABTS•^+^ was equivalent to that of VC standard in the same concentration range. In the concentration extent of 0−1.0 mg/mL, the inhibition percentage value of FSI extract increased gradually, but which was lower than that of RU and VC. At high concentration (1.0 mg/mL), FSI extract, RU, and VC had the same ABTS•^+^ scavenging effect. To sum up, the outcomes indicated that the FSI extract had a moderate ABTS•^+^ scavenging capacity and can be used as a potential antioxidant in the range of experimental concentration.

#### 2.4.3. Scavenging Capacity of Superoxide Anion (SCSA) Radical

Figure 8C shown the SCSA of the FSI extracts compared with the positive control (VC and RU). The inhibition capacity (y) and concentration (x) of the FSI extract was associated with the following second-order equations, respectively: y = 41.464x^2^ + 12.29x + 2.9903 (*R^2^* = 0.9990). The positive control (VC and RU) and FSI extracts possessed the inhibitory ability of superoxide anion through dose-dependent ways. The order of SCSA of all samples as follows: VC > RU > FSI. At the tested concentrations (0.00–1.00 mg/mL), VC had the best antioxidant capacity, and the value on SCSA of FSI was up to 56.61% at 1.0 mg/mL. In short, these results suggested that the FSI extract had a general activity for SCSA in the range of experimental concentration.

#### 2.4.4. Ferric Reducing/Antioxidant Power

FRAP is a vital index, which can evaluate the potential antioxidant activity of active extracts [38]. The Fe^2+^ reducing power (y) of the FSI extract is positively related with concentration (x) (*p* <0.05), showing a quadratic relationship. For FSI extract, the equation takes the form y = −6.25x^2^ + 25.45x + 62.3 (*R^2^* = 0.9941). It was obvious that the FRAP of FSI extracts and the positive control (VC and RU) were dose-dependent and went up with the growth of sample concentration in the extent of the tested concentrations, and the growth trend was consistent (Figure 8D). The order of all samples on FRAP was as follows: VC > RU > FSI. In the range of concentration, the inhibition of FSI was up to 81.4%, with good reduction capacity.

## 3. Materials and Methods

### 3.1. Sample of FSI

FSI was purchased from double season *Sophora japonica L*. (Xia county, Yuncheng, Shanxi province, China) and was naturally dried. The pulverized samples were screened (60–80 mesh). In addition, the processed FSI materials were stored under normal temperature, dry, and light-proof conditions for future use.

### 3.2. Chemicals and Apparatus

Seven authentic reference standards—rutin (≥98%), narcissoside (≥98%), isorhamnetin (≥98%), kaempferol (≥98%), quercetin (≥98%), and nicotiflorin (kaempferol-3-O-rutinosid, ≥98%) were all obtained from J&K chemical Technology Co., Ltd. (Beijing, China). HPLC-grade phosphoric acid and acetonitrile were bought from Tianli Chemical Reagent Co., Ltd. (Tianjin, China) and Oceanpak (Goteborg, Sweden). The deionized water used in the experiment was obtained by the Milli-Q water purification system (Millipore, Bedford, MA, USA). DPPH was acquired from SIGMA-ALDRICH-INC. ABTS•^+^ and Tris were purchased from Solarbio Sciences &Beijing Technology Go., Ltd. (Beijing, China). Other chemicals and reagents were of analytical grade, and were purchased from Tianjin Guangfu Fine Chemical Research Institute (Tianjin, China).

### 3.3. Preparation of Standard Solution

For UV: Accurately added 20 mg rutin authentic reference standard to 100 mL volumetric flask with 90% (*v/v*) ethanol (0.2 mg rutin per 1 mL).

For HPLC: The mixed-standard stock solution was deployed by adding accurately 1 mg of six reference standards to a 10 mL volumetric flask, and dilute with methanol to volume, and mix. Gradient work solutions were prepared via the dilution of the mixed-standard stock solution with methanol.

All the solutions were stored in a 4 °C refrigerator before use.

### 3.4. Determination of TFFSI by UV

The content of total flavonoid in FSI was measured by a colorimetric determination according to Heimler, D, et al. [40] with slight modifications in the study. One milliliter of the suitably diluted sample, 5 mL deionized water, and 1 mL 5% NaNO_2_ solution were added, incubated for 6 min; then 1 mL freshly prepared 10% Al(NO_3_)_3_ solution was mixed, incubated for 6 min; finally 10 mL of 1 mol/L sodium hydroxide solution was added. The final volume was added to 25 mL with deionized water. The mixture was kept at room temperature for 15 min, after which its absorbance value was read at 511 nm with an ultraviolet spectrophotometer (UV−5500PC, METASH, Shanghai, China) against the mixture without the sample. The content of total flavonoids was calculated by the calibration curve of rutin (rutin g/g sample). The regression data was listed in Table 3.

### 3.5. Determination of Six Main Flavonoids by HPLC

**Figure 9:**
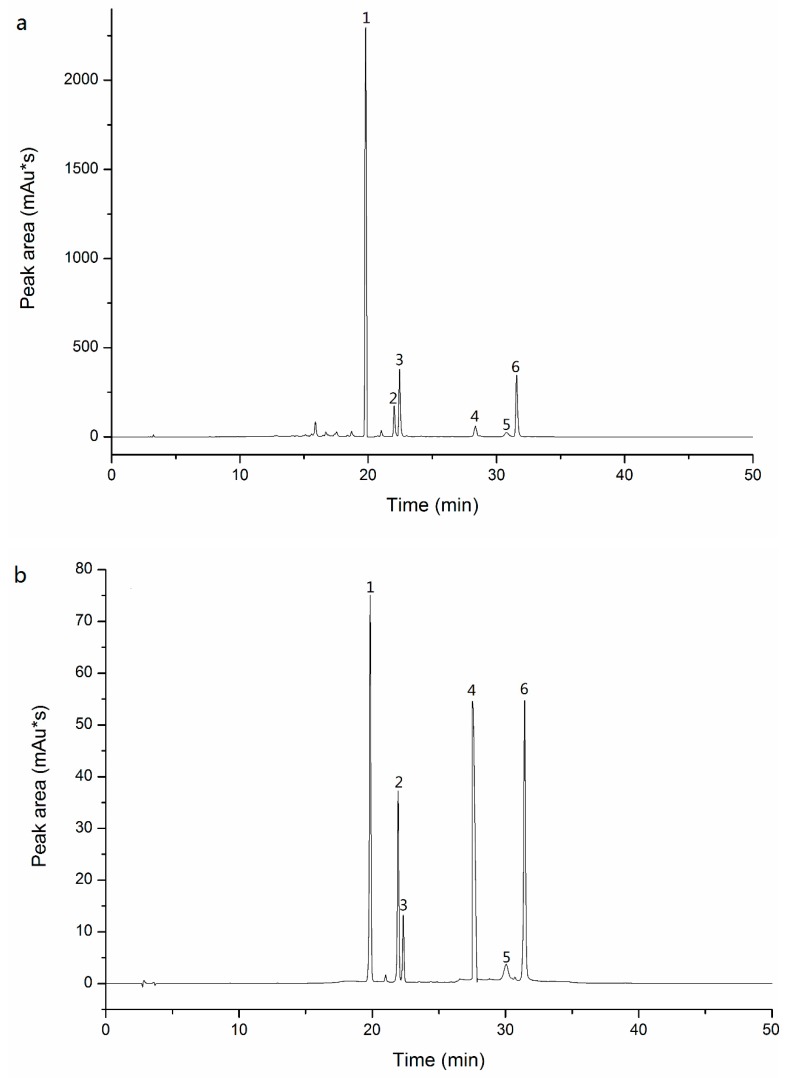
The HPLC chromatograms of FSI samples (**a**) and standards mixture (**b**) detected at wavelength 360 nm. 1: RU; 2: NI; 3: NA; 4: KA; 5: IS; and 6: QU.

Quantifications of six flavonoids extracted from FSI were analyzed through an Agilent 1260 HPLC system (Agilent Technologies, Santa Clara, CA, U.S.) with a Diamonsil TM C18 column (250 mm by 4.6 mm, 5μm, Dikma, Beijing, China). The mobile phase was comprised of 0.1% (*v/v*) H_3_PO_4_ aqueous solution (A) and acetonitrile (B) which was filtered by a 0.45 μm microporous membrane and degassed by ultrasound before use. The column temperature was set at 30 °C, the injection volume was 5 μL and the flow rate was 1.0 mL/min. The detection wavelength was 360 nm. The gradient elution conditions were as follows: 0–30 min, 95%–65% A; 30–35 min, 65%–95% A; and 35–50 min, 95% A. Flavonoids of FSI were confirmed by the comparison of the UV spectra and relative retention time of sample and standards (Figure 9). Respective calibration curves were used to quantify the content of flavonoids. Results were represented as g/g*100%. The regression data for RU, NI, NA, KA, IS, and QU were listed in Table 3.

### 3.6. Sample Preparation Based on UAE

Accurately add 1 g FSI sample to a 50 mL centrifuge tube; perform ultrasonic extraction (Ultrasonic Cleaner SB−5200DT, 240 W, 40 kHz, Ningbo Scientz Biotechnology Co., LTD, Ningbo, China) with 15 mL 70% ethanol at 60 °C for 30 min. After centrifugation (4500 r/min, 15 min, Low-speed centrifuge SC−3614, Anhui USTC Scientific Instruments Co.,Ltd, Hefei, China), the liquid supernatant was transferred. The extraction was performed three times given the yield and cost, and the extracts were filtered by filter membrane (0.45 μm, Tianjin jinteng experimental equipment Co. Ltd, Tianjin, China) before testing in HPLC. All experiments were done in triplicate. The extraction rate was calculated as follows: Extraction rates = Content of flavonoids extracted by certain parameters/ Actual contents of FSI flavonoids×100%.

### 3.7. Experimental Design

#### 3.7.1. Univariate Experiments Design

The very important parameters affecting the extraction rates of flavone from FSI were the type of organic solvent, the concentration (*v/v*) of extraction solvent, the species of surfactants added in extraction solvent, the liquid/solid ratio (mL/g), ultrasound extraction time (min), and ultrasound extraction temperature (°C). The extraction rates of TFFSI and six main flavonoids were determined to select their optimal points by changing a single condition and fixing other conditions in this section.

#### 3.7.2. Box–Behnken Design

Box–Behnken design (BBD) is a kind of (nearly) rotatable second-order design according to three-level incomplete factorial design [41]. After univariate experiments, the factors of extraction procedure were optimized employing three-level, four-factor BBD and RSM. Four main independent variables, the ethanol concentration, liquid/solid ratio, ultrasound extraction time, and ultrasound extraction temperature were defined as A, B, C, and D, respectively. Moreover, the range of values was depended on the outcome of the above experiments. The dependent variables were the extraction rates of TFFSI (Y_1_), RU (Y_2_), NI (Y_3_), NA (Y_4_), KA (Y_5_), IS (Y_6_), and QU (Y_7_), which were used for determining the optimal conditions. There was a sum of 29 runs based on BBD including five center points conducted in out of order. The detailed experimental design was listed in Table 5.

### 3.8. Assess the Antioxidant Activity of Hydroalcoholic Extracts from FSI

#### 3.8.1. Sample Preparation

The optimal extraction method in 3.7 was adopted to obtain the total flavonoids in FSI, and the extraction solution was combined, use a rotary evaporator (RE−52A, Shanghai, China) to concentrate the extracts, then placed in the −60 °C refrigerator (Haier, Qingdao, China) overnight. A vacuum freeze dryer (SCIENTZ−12N, Ningbo, China) was used to dry the powder. The dry extracts were stored hermetically in dark, airy, and dry environment before the experiment. When used, it was configured as a gradient sample solution.

In the antioxidant activity test, hydroalcoholic extracts from FSI, VC standard (positive reference control), and rutin standard (positive reference control) were all prepared into 0.2, 0.4, 0.6, 0.8, and 1.0 mg/mL gradient solution to be tested.

#### 3.8.2. Measurement of Scavenging Capacity on DPPH Radicals

The scavenging capacity of the hydroalcoholic extracts from FSI on DPPH radicals was assessed basing on the methods of Yan-Hwa Chu [42] and L.M. Cheung [43] with minor modifications.

5 mL of 0.2 mmol/L DPPH radical solution (configured with anhydrous ethanol and stored away from light) was added into a test tube with 1 mL of VC standard, rutin standard, or FSI hydroalcoholic extract of gradient concentrations (0–1.0 mg/mL). Anhydrous ethanol was substituted for the sample as a control, and recorded as A_control_. The mixture was reacted at room temperature and the absorbance (Abs) was monitored after mixing 30 min by determining at 517 nm with a UV and recorded as A_sample_. The percentage of inhibition on DPPH radicals was computed as follows: Inhibition%= (A_control_- A_sample_)/ A_control_×100.

#### 3.8.3. Measurement of Scavenging Activity on ABTS•^+^ Radicals

The ABTS•^+^ assay was conducted according to the methods of Kriengsak Thaipong et al. [44] with minor modifications. In short, 7.0 mmol/L of ABTS•+ was blended with 1.4 mmol/L of K_2_S_2_O_8_ in equal quantities. The mixture was placed from light for 16 h to form ABTS•^+^ working solution at room temperature (12–16 °C). Before testing, the blue–green ABTS•^+^ solution was diluted with 100% alcohol until the absorbance value of 0.70 ± 0.02 at 734 nm, and recorded as A_control_. Then, 0.2 mL of VC standard, rutin standard, or FSI hydroalcoholic extract sample were mixed with 5 mL of ABTS•^+^ solution. The absorbance was observed at 734 nm after 6 min, and recorded as A_sample_. The percentage of inhibition on ABTS•+ was measured as DPPH.

#### 3.8.4. Superoxide Anion Scavenging Activity (SASA)

Superoxide anion radical is weak oxidizer. SASA was determined by pyrogallol autoxidation, which had characteristic absorption peak at 320 nm. When antioxidants were added, the oxidation process was inhibited and the solution produced a subtractive effect at the maximum absorption wavelength. The literatures indicated that the method could be effectively used to detect the antioxidant capacity of VC, rutin, and flavonoids [45].

The scavenging ability on SASA was assessed by the methods of Xican Li [38] with some modifications. Briefly, 1 mL the sample solution was mixed with 4.5 mL Tris-HCl buffer (0.05 mol/L, pH 8.2), adding 0.5 mL pyrogallol (3 mmol/L), then mixed rapidly at room temperature, reacting for 5 min, and finally, 1.0 m L of 8 mol/L HCl was added to end the reaction. The absorbance at 320 nm of the solution was read against the Tris-HCl buffer, and recorded as A_sample_. The reaction solution without adding sample was marked as the control, and recorded as A_control_. The percentage of inhibition on •O_2_^-^ was calculated as DPPH.

#### 3.8.5. Ferric Reducing/Antioxidant Power

The reducing power of the extracts were performed spectrophotometric ally at 700 nm. The higher the absorbance value of the reaction mixture, the stronger reducing power. The reducing power of FSI extracts was measured, referring to the methods of Yan-Hwa Chu [42] with modifications. One milliliter FSI extracts, 1 mL phosphate buffer (0.2 mol/L, pH 6.6), and 1.0 mL of K_3_[Fe(CN)_6_] (1%) were added to the centrifuge tube, and the mixture was set at 50 °C water bath for 20 min. After rapid cooling, 1 mL C_2_HCl_3_O_2_ (10%) was added into the mixture, after full reaction, standing 2.0 mL the supernate was mixed with 3.0 mL distilled water and 0.2 mL FeCl_3_ (0.1%), incubated for 10 min from light at room temperature, and read the absorbance values by UV at 700 nm immediately, and recorded as A_sample_. The absorbance values of the mixture that distilled water was substituted for the flavonoids sample solution at 700 nm, and recorded as A_control_. The percentage of inhibition on FRAP was calculated as DPPH.

Each program was measured in triplicate and the data were calculated as mean and standard deviation (*n* = 3).

### 3.9. Statistical Analysis

All experiments were operated in triplicate, and the results were measured as mean ± standard deviation (SD). A Design-Expert 8.0.6 program (Stat-Ease Inc., Minneapolis, USA) was applied for experimental design and statistical analysis. The significance and interaction of parameters was analyzed by the analysis of variance at the RSM experiment, and the difference (*p* <0.05) was considered significant. The significance test of the model was conducted, and we analyzed the effect of each parameter about the extraction rates of TFFSI and six flavonoids, and response surfaces were presented as a three-dimensional image.

## 4. Conclusion

All told, our study developed the combination of ultrasound and RSM extraction method, used for the efficient and green extinction of RU, NI, NA, KA, IS, and QU from FSI. After single-factor experiments, we investigated the six important extraction conditions affecting the extraction rate of flavonoids. Then we optimized four parameters simultaneously, and also demonstrated the interaction between the parameters by RSM. On that basis, we obtained the optimal process parameters of the highest extraction rates of TFFSI and six flavonoids from FSI. The optimal conditions were: The ethanol concentration 70%, liquid–solid ratio 15.30 mL/g, ultrasound time 30 min, and ultrasound temperature 61 °C. The actual extraction rates of TFFSI, RU, NI, NA, KA, IS, and QU were 26.4260%, 14.6101%, 2.9310%, 7.1987%, 0.1041%, 0.4920%, and 2.7998%, respectively, with RSD values less than 0.92%. The present study performed an HPLC method with simple and rapid, being appropriate for the simultaneous detection of flavonoids in FSI extracts.

The antioxidant activity assessment results revealed that the antioxidant ability of the FSI hydroalcoholic extracts was concentration-dependent, which had stronger inhibition of DPPH radical, ABTS•^+^, •O_2_^−^, and FRAP at higher concentrations. So, flavonoids could make an important contribution to the antioxidant capacity in FSI extract. After determining the antioxidant activity of FSI extract, the pharmacological properties of the antioxidants will be further studied. The efficient radical scavenging ability in FSI extracts shows the viability of the extraction process in recovering and utilizing value-added bioactive substances from natural products.

## Figures and Tables

**Table 1:** Extraction conditions of different extraction methods in Flos Sophorae Immaturus (FSI).

No.	Flavonoids (Extraction Yield (%))							Method	Solvent	Ratio mL/g	Time	Temperature (°C)	Literature
	Rutin	Nicoti- Florin	Narci- Ssoside	Kaem-Pferol	Isorham-Netin	Querc-Etin	Genistein						
**1**	11.68	1.50	2.39	0.31	0.33	2.76	-	Microwave	deep eutectic solvent	26	20 m	62	Gen Wang, 2018 [15]
**2**	32.11	-	-	0.06	0.17	6.26	0.002	Microwave	100% methanol	50	80 s	Unclear	Jin-Liang Liu, 2016 [16]
**3**	20.86	0.43	2.59	-	-	-	-	Ultrasound	82% methanol	56	60 m	Unclear	Zhisheng Xie, 2014 [17]
**4**	13.38		1.98			0.55		Ultrasound	80% methanol	500:3	30 m	30	Zhisheng Xie, 2014 [21]
**5**	25.26	-	-	-	-	-	-	Infrared	70% methanol	30	4.8 m	Unclear	Fa-jie Li, 2011 [18]
**6**	10.28							FIASE	Methanol	25	6 m		ZhibinGan, 2011 [19]
**7**	23.59							Water bath	1.72 mol/L 1-butyl-3-methylimidazoliumchloride	75	20 m	50	Jianlin Huang, 2010 [25]
**8**	21.1					0.62		Ultrasound	Methanol	50	30 m	60	Jingjing Xu, 2007 [26]

**Table 2:** Analysis of variance (ANOVA) statistics of the regression model for the extraction rates of RU, NI, NA, KA, IS, QU, and the TFFSI.

Variables	TFFSI		RU		NI		NA		KA		IS		QU	
	*F*-Value	*p*-Value	*F*-Value	*p*-Value	*F*-Value	*p*-Value	*F*-Value	*p*-Value	*F*-Value	*p*-Value	*F*-Value	*p*-Value	*F*-value	*p*-Value
Model	133.35	<0.0001	48.48	<0.0001	70.29	<0.0001	662.95	<0.0001	112.16	<0.0001	79.43	<0.0001	690.27	<0.0001
A	17.38	0.0009	4.33	0.0564	22.94	0.0003	52.04	<0.0001	15.35	0.0015	0.41	0.5305	160.99	<0.0001
B	156.13	<0.0001	0.00008	0.9928	0.59	0.4569	16.22	0.0012	64.38	<0.0001	49.18	<0.0001	6.09	0.0271
C	34.55	<0.0001	2.16	0.1636	15.29	0.0016	60.81	<0.0001	19.56	0.0006	5.26	0.0378	6.15	0.0265
D	54.73	<0.0001	0.36	0.5600	106.87	<0.0001	568.71	<0.0001	51.42	<0.0001	24.77	0.0002	18.99	0.0007
AB	3.24	0.0936	23.43	0.0003	0.41	0.5317	0.29	0.6006	15.42	0.0015	4.30	0.0570	0.0074	0.9328
AC	45.36	<0.0001	2.89	0.1111	5.82	0.0302	19.49	0.0006	3.32	0.0899	0.50	0.4898	2.75	0.1198
AD	6.30	0.0250	0.045	0.8352	0.00915	0.9252	8.22	0.0124	6.10	0.0270	8.30	0.0121	0.15	0.7024
BC	176.26	<0.0001	24.28	0.0002	2.04	0.1751	6.51	0.0231	2.82	0.1153	32.78	<0.0001	26.70	0.0001
BD	25.02	0.0002	8.46	0.0114	0.98	0.3379	0.91	0.3557	152.88	<0.0001	0.066	0.8003	121.18	<0.0001
CD	44.90	<0.0001	8.54	0.0111	20.25	0.0005	74.23	<0.0001	21.86	0.0004	5.09	0.0406	613.28	<0.0001
A^2^	323.61	<0.0001	200.73	<0.0001	191.76	<0.0001	2462.20	<0.0001	648.50	<0.0001	373.58	<0.0001	3626.45	<0.0001
B^2^	866.73	<0.0001	361.20	<0.0001	184.16	<0.0001	2429.41	<0.0001	288.60	<0.0001	502.16	<0.0001	4196.71	<0.0001
C^2^	66.60	<0.0001	170.37	<0.0001	152.27	<0.0001	2085.05	<0.0001	654.16	<0.0001	428.39	<0.0001	3500.95	<0.0001
D^2^	555.98	<0.0001	194.72	<0.0001	637.69	<0.0001	5760.41	<0.0001	232.85	<0.0001	197.93	<0.0001	2151.17	<0.0001
Lack of fit	0.68	0.7197	0.57	0.7879	0.19	0.9859	0.38	0.9041	0.41	0.8828	0.44	0.8691	0.89	0.6025
*R^2^*	0.9926		0.9798		0.9860		0.9985		0.9912		0.9876		0.9986	
Adjusted *R^2^*	0.9851		0.9596		0.9719		0.9970		0.9823		0.9751		0.9971	

**Table 3:** Regression data, the limit of quantifications (LOQs, and the limit of detections (LODs) for TFFSI and six components.

Compound	Calibration Curve	R^2^	Linearity Range/(mg/mL)	LOD/(mg/mL) ^c^	LOQ/(mg/mL) ^d^
**The total flavonoids of Flos Sophorae Immaturus ^a^**	Y = 9.93278X + 0.01922	0.99796	0.004–0.100	0.0030	0.0040
**Rutin ^b^**	Y = 9759.82662X−1.23991	0.99994	0.003125–1.000	0.0020	0.0030
**Nicotiflorin ^b^**	Y = 2299.7295X + 19.66392	0.99162	0.006–2.000	0.0050	0.0055
**Narcissoside ^b^**	Y = 2206.89713X + 67.81067	0.9973	0.004147–1.6	0.0030	0.0041
**Kaempferol ^b^**	Y = 21654.77389X + 139.37839	0.99905	0.004–1.000	0.0030	0.0038
**Isorhamnetin ^b^**	Y = 10605.6954X−30.38794	0.99682	0.00816–0.1700	0.0070	0.0080
**Quercetin ^b^**	Y = 12930.14145X + 159.16393	0.99631	0.0045–1.2000	0.0040	0.0042

^a^ 7 different concentrations (*n* = 10); Y: The UV absorbance value of analyte; X: The concentration of rutin (mg/mL), ^b^ 7 different concentrations (*n* = 10); and Y: The peak area of compound; X: The concentration of compound (mg/mL), ^c^ LOD (the limit of detection), and ^d^ LOQ (the limit of quantification).

**Table 4:** Precision, repeatability, and recovery yields of the total flavonoids of Flos Sophorae Immaturus and six compounds (*n* = 6).

Analyte	Intra-Day RSD			Inter-Day RSD			RSD			AmountAdded(mg)	Recovery(%)	RSD(%)
	UVv ^a^ (%)	Pa ^b^ (%)	Rt ^c^ (%)	UVv (%)	Pa (%)	Rt (%)	UVv (%)	Pa (%)	Rt (%)			
**The total flavonoids of Flos Sophorae Immaturus**	2.33	-	-	2.46	-	-	3.21	-	-	0.020	98.93	2.51
										0.040	98.90	2.68
**Rutin**	-	0.40	0.16	-	0.89	0.22	-	0.19	0.20	0.020	99.30	2.65
										0.040	98.70	2.14
**Nicotiflorin**	-	0.50	0.70	-	0.67	0.10	-	0.44	0.04	0.020	99.41	2.31
										0.040	98.77	2.47
**Narcissoside**	-	0.13	0.05	-	0.24	0.10	-	0.04	0.04	0.020	97.98	2.41
										0.040	99.12	2.84
**Kaempferol**	-	0.31	0.20	-	0.47	0.21	-	0.21	0.30	0.020	99.21	2.36
										0.040	97.87	3.10
**Isorhamnetin**	-	0.32	0.11	-	0.41	0.09	-	0.18	0.10	0.020	98.68	2.98
										0.040	99.21	3.16
**Quercetin**	-	0.89	0.55	-	0.95	0.62	-	0.91	0.60	0.020	97.94	2.98
										0.040	97.68	2.78

**^a^**: Ultraviolet value, ^b^: Peak area, and ^c^: Retention time.

**Table 5:** Results of the Box–Behnken design (BBD) for the extraction rates of RU, NI, NA, KA, IS, QU, and the TFFSI.

Runs	Factors				Extraction Rate (%)						
	A (E ^a^/%)	B (L ^b^/mL/g)	C (t ^c^/min)	D (T ^d^/°C)	Y_1_	Y_2_	Y_3_	Y_4_	Y_5_	Y_6_	Y_7_
1	0(70)	1(20)	0(30)	−1(55)	23.05	11.80	2.25	5.06	0.0095	0.2846	0.2846
2	0	−1(10)	−1(25)	0(60)	24.31	11.51	2.61	5.96	0.0060	0.1655	0.9456
3	1(75)	−1	0	0	22.62	12.53	2.59	5.89	0.0054	0.1865	0.9224
4	0	−1	1(35)	0	22.54	12.89	2.48	5.70	0.0074	0.2325	1.1910
5	1	0(15)	0	1(65)	23.67	12.50	2.54	5.76	0.0394	0.3166	1.1283
6	0	0	−1	−1	24.28	12.75	2.41	5.42	0.0124	0.2426	1.8381
7	0	0	0	0	26.67	14.91	2.85	7.25	0.1022	0.4938	2.8469
8	0	0	0	0	26.27	14.42	2.90	7.21	0.1042	0.4880	2.8669
9	0	−1	0	1	22.34	11.98	2.49	5.72	0.0049	0.2464	1.3541
10	1	0	1	0	25.49	12.67	2.60	5.87	0.0054	0.2497	0.99661
11	0	1	−1	0	23.36	12.38	2.59	5.81	0.0170	0.2967	1.1399
12	0	0	0	0	26.27	14.42	2.89	7.15	0.0922	0.4898	2.8969
13	1	1	0	0	23.98	11.77	2.57	5.76	0.0477	0.2886	0.9891
14	0	0	0	0	26.27	14.42	2.95	7.28	0.1102	0.5038	2.7947
15	0	1	1	0	25.75	11.92	2.56	5.74	0.0354	0.1871	1.0083
16	0	0	1	1	25.65	12.83	2.52	5.73	0.0531	0.2814	1.8018
17	−1(65)	0	1	0	24.75	12.57	2.45	5.59	0.0064	0.2151	1.3006
18	−1	−1	0	0	23.25	11.46	2.46	5.71	0.0076	0.2178	1.1868
19	0	−1	0	−1	22.65	12.43	2.28	5.12	0.0438	0.2062	1.0557
20	0	0	−1	1	24.07	12.36	2.44	5.58	0.0102	0.3369	0.8409
21	1	0	−1	0	23.68	12.90	2.59	5.87	0.0051	0.2580	1.0143
22	−1	0	0	1	24.52	12.37	2.41	5.45	0.0185	0.2792	1.4327
23	0	0	1	−1	23.76	12.14	2.17	4.91	0.0081	0.2567	0.9920
24	−1	1	0	0	24.05	12.50	2.49	5.62	0.0103	0.2559	1.2598
25	−1	0	0	−1	23.43	12.46	2.22	5.05	0.0132	0.2864	1.4970
26	0	0	0	0	26.27	14.42	2.99	7.19	0.0992	0.4514	2.8688
27	−1	0	−1	0	25.06	12.17	2.61	5.93	0.0011	0.2453	1.1978
28	1	0	0	−1	23.36	12.51	2.34	5.14	0.0092	0.2349	1.2211
29	0	1	0	1	24.30	12.43	2.53	5.59	0.0953	0.3169	1.0328

^a^ The ethanol concentration (*v/v* %), ^b^ the liquid/solid ration (mL/g), ^c^ ultrasound extraction time (min), and ^d^ ultrasound extraction temperature (°C).
